# Simple and validated method to quantify lacosamide in human breast milk and plasma using UPLC/MS/MS and its application to estimate drug transfer into breast milk

**DOI:** 10.1186/s40780-023-00295-w

**Published:** 2023-09-01

**Authors:** Ayako Furugen, Ayako Nishimura, Takeshi Umazume, Hina Ishikawa, Katsuya Narumi, Masaki Kobayashi

**Affiliations:** 1 Laboratory of Clinical Pharmaceutics & Therapeutics, Division of Pharmasciences, Faculty of Pharmaceutical Sciences, Hokkaido University, Kita-12-Jo, Nishi-6-Chome, Kita-Ku, Sapporo, 060-0812 Japan; 2 Department of Pharmacy, Hokkaido University Hospital, Kita-14-Jo, Nishi-5-Chome, Kita-Ku, Sapporo, 060-8648 Japan; 3 Department of Obstetrics, Hokkaido University Hospital, Kita-14-Jo, Nishi-5-Chome, Kita-Ku, Sapporo, 060-8648 Japan

**Keywords:** Breast milk, Lacosamide, Milk to plasma ratio, Relative infant dose, UPLC/MS/MS

## Abstract

**Background:**

Epilepsy is a common neurological disorder. Lacosamide is a third-generation antiepileptic drug used to treat partial-onset seizures. Limited information is currently available on the transfer of lacosamide to breast milk. To facilitate studies on the safety of lacosamide use during breastfeeding, we aimed to develop a method to quantify lacosamide in human breast milk and plasma using ultra-performance liquid chromatography/tandem mass spectrometry.

**Methods:**

Fifty microliters of breast milk or plasma was used, and samples were prepared by protein precipitation using methanol containing lacosamide-d_3_ as an internal standard (IS). Chromatography was performed using an ACQUITY HSS T3 column with an isocratic flow of 10 mM ammonium acetate solution/methanol (70:30, v/v). Lacosamide and IS were detected by multiple reaction monitoring in positive ion electrospray mode. The run time was 3.5 min.

**Results:**

Calibration curves were linear and in the range of 0.5 to 100 ng/mL both in breast milk and plasma. The validation assessment indicated that precision, accuracy, matrix effects, selectivity, dilution integrity, and stability were acceptable. The developed method was successfully applied to quantify lacosamide in breast milk and plasma obtained from a volunteer who had been orally administered lacosamide twice a day (100 mg × 2). Relative infant dose of lacosamide was estimated to be 14.6% in breast milk at five time points.

**Conclusions:**

We developed a simple and robust method to quantify of lacosamide in human breast milk and plasma. This method could be useful for in future studies investigating the safety of lacosamide use during breastfeeding.

## Background

Breastfeeding is recommended owing to its multiple health benefits for both mothers and breastfed infants [1]. However, the benefits and risks associated with medicines administered during breastfeeding in breastfed infants must be considered. Although medications are commonly used in lactating women in clinical practice [2], detailed data of drug transfer into breast milk remains to be available in some cases. Therefore, investigation of drug transfer into breast milk and estimation of exposure to breastfed infants are important to better understand and predict the risks during breastfeeding. Parameters such as milk/plasma (M/P) ratio and relative infant dose (RID) are widely used to estimate drug transfer to breast milk and exposure to infants. Further, RID is a useful comparative parameter to assess the amount of drugs received by infants via breast milk [3].

Epilepsy is a common neurological disorder worldwide. Approximately 50% of women with epilepsy are of childbearing age [4]. Recent studies have assessed the transfer of antiepileptic drugs into breast milk and the safety associated with this during breastfeeding [4, 5]. Levetiracetam and lamotrigine are the most commonly prescribed antiepileptic drugs for pregnant or lactating women with epilepsy [5]. Further, lacosamide is a third-generation antiepileptic drug that selectively enhances the slow inactivation of voltage-gated sodium channels [6]. Lacosamide was first introduced in clinical practice in 2008 as an adjunctive treatment for adults with focal-onset seizures, with or without generalization. Currently, it is clinically used as a monotherapy for focal onset seizures in patients [7]. In general, newer antiepileptic drugs have more favorable pharmacokinetic profiles and fewer drug interactions compared with those of old drugs. Accumulating information on lacosamide use during pregnancy and breastfeeding can lead to broader therapeutic options for women with epilepsy. Only a few case reports on lacosamide use during breastfeeding are currently available [8]. Ylikiotlia et al. reported that lacosamide level was low in breast milk 5 days after delivery, and RID value was estimated to be 1.8% [9]. Zarubova et al. reported that breast milk levels 20 days after delivery were 14.27 μM, 21.8 μM, and 16.92 μM before taking the regular lacosamide dose, 2 h, and 6 h after the administration, respectively [10]. Kohn et al. indicated in their case report that M/P ratio of lacosamide was 0.5 and RID value 22% [11]. Furthermore, they reported that the health and development of the infant assessed over a telephone call were normal at 6 months of age. Monfort et al. reported that the RID value of lacosamide was 29.9% [12]. Landmark et al. reported that breast milk levels were 21 μM 5 days after delivery at 12 h after administration and 25 μM 5weeks after delivery at 13 h after administration [13]. They reported an M/P ratio of 0.83. Although information on the use of lacosamide during breastfeeding is increasing, its safety during breastfeeding has not been fully evaluated. To facilitate further studies on the permeability and safety of lacosamide, a simple and robust method is required that quantifies breast milk and plasma.

Several methods have been developed to measure lacosamide levels in plasma (humans and rats) using high-performance liquid chromatography-ultraviolet (HPLC–UV) [14], liquid chromatography, or ultra-performance liquid chromatography tandem mass spectrometry (LC/MS/MS or UPLC/MS/MS) [15–24]. However, only a few methods are available for quantifying lacosamide in human breast milk [25]. Notably, LC/MS/MS and UPLC/MS/MS are tools widely used for analyzing drug concentrations. Since breast milk is a complex matrix, a robust and validated method is required to accurately quantify clinical samples. In particular, matrix effects should be considered in mass spectrometric methods. Recently, Monfort et al. developed an LC/MS/MS method for analyzing 19 analytes (17 drugs and 2 active metabolites), including lacosamide [25]. They demonstrated the suitability of the method for quantifying vortioxetine and clomiphene in human breast milk and revealed the pharmacokinetic parameters of these drugs. The method focuses on lacosamide in human breast milk and plasma, and its application is unavailable.

In this study, we aimed to establish a simple and robust method using UPLC/MS/MS to quantify lacosamide levels in human breast milk and plasma. Furthermore, we applied this developed and validated method to quantify lacosamide in breast milk and plasma donated by a breastfeeding woman who had been prescribed the medication and estimated the M/P and RID.

## Methods

### Reagents

Lacosamide and its deuterated form (lacosamide-d_3_) were purchased from Toronto Research Chemicals (Toronto, ON, Canada). Next, HPLC-grade methanol was obtained from FUJIFILM Wako Pure Chemical Corporation (Osaka, Japan). An HPLC-grade aqueous ammonium acetate solution was purchased from Nacalai Tesque (Kyoto, Japan). Pooled breast milk was obtained from LEE BioSolutions (Maryland Heights, MO, USA) for method validation. Pooled plasma samples from healthy human donors were obtained from Cosmo Bio (Tokyo, Japan). To investigate the matrix effect, breast milk samples from six donors were obtained from LEE BioSolutions. Individual human plasma samples from six healthy female donors were obtained from Cosmo Bio.

### Calibration curve

Standard and internal standard (IS) stock solutions were prepared using methanol. Concentrations of the standard stock solutions in methanol were 40, 80, 200, 400, 800, 2,000, 4,000, and 8,000 ng/mL. These stock solutions were stored at − 80 °C. Calibration samples were prepared immediately before analysis by appropriately diluting the stock solutions in blank breast milk or plasma. Specifically, the calibration samples were made by spiking 200 μL of blank pooled breastmilk (LEE BioSolutions) or plasma (Cosmo Bio) with 2.5 μL of each stock solution. Fifty microliters of the calibration sample was used for the procedures described in Sample preparation. Calibration curves of eight concentrations (0.5, 1.0, 2.5, 5.0, 10, 25, 50, and 100 ng/mL) were obtained by plotting the peak area ratio (lacosamide/IS) against the theoretical concentration and were fitted using least-squares regression with 1/x weighting.

### Sample preparation

To 50 μL of breast milk or plasma samples, 200 μL of methanol containing the IS (1 ng/mL) was added and mixed by vortexing. The mixture was centrifuged at 13,000 × *g* for 15 min at 4 °C. Subsequently, 100 μL of the supernatant was carefully collected and filtered through a DISMIC-13HP filter (0.2 μm, ADVANTEC, Tokyo, Japan). Next, 2 μL of the sample was injected into the UPLC/MS/MS.

### UPLC/MS/MS

Ultra-performance liquid chromatography (ACQUITY UPLC H-Class, Waters) and an ACQUITY UPLC HSS T3 column (2.0 × 50 mm, 1.8 µm, Waters) were used for chromatographic separation. An in-line column filter kit (Waters) was used to protect the column. The column temperature was set at 40 °C. The mobile phase was an isocratic flow of methanol/10mM ammonium acetate (30:70, v/v) at a flow rate of 0.4 mL/min. The total runtime was 3.5 min. Positive-ion electrospray tandem mass spectrometric analysis was performed using a Xevo TQ-S (Waters) with multiple reaction monitoring (MRM). Transitions *m/z* 251.2 → 108.0 for lacosamide and *m/z* 254.0 → 108.0 were monitored for quantification. As a qualifier ion, *m/z* 251.2 → 91.0 was also monitored. The dwell time for each ion was 200 ms/ion. The cone was set at 20V, and the collision energies were 6, 6, and 18V for the lacosamide, IS, and lacosamide qualifier, respectively. Data were analyzed using TargetLynx software.

### Method validation

Method validation was performed according to the Food and Drug Administration (Guidance for Industry: Bioanalytical Method Validation 2018) and European Medicines Agency (Guideline on Bioanalytical Method Validation 2011) guidelines.

Intra-day precision and accuracy were investigated by analyzing six samples at four concentrations (0.5, 1.25, 12.5, and 80 ng/mL) on the same day. Inter-day precision and accuracy were assessed by analyzing the samples at four concentrations (0.5, 1.25, 12.5, and 80 ng/mL) on six days. The samples were processed as described in Sample preparation and measured. The relative error (R.E.) was calculated as [(measured concentration − nominal concentration)/nominal concentration] × 100 (%). The precision was defined as the relative standard deviation (R.S.D.). The lower limit of quantification (LLOQ) was determined using precision and accuracy data and a signal-to-noise (S/N) ratio > 10. The accuracy and precision should be <  ± 15%, except for LLOQ, for which the acceptable limit is <  ± 20%.

To investigate selectivity, six different lots of matrix (human breast milk or plasma) with or without the LLOQ (0.5 ng/mL) of lacosamide were prepared and analyzed. The interference peak detected at the retention time of lacosamide should be < 20% of the LLOQ and 5% of IS. Carry-over was investigated by injecting a blank matrix (human breast milk or plasma) after injecting the highest concentration of the sample (100 ng/mL). The area responses of the blank matrices were compared with those of the LLOQ. Further, the peak area of the blank samples after injecting the highest concentration should not be over 20% of the peak area at LLOQ and 5% of the peak area of the IS.

To assess matrix effects, the accuracy and precision of multiple lots from different donors were determined. Six lots of breast milk or plasma were spiked with lacosamide at three concentrations (1.25, 12.5, and 80 ng/mL). The samples were processed as described in Sample preparation and measured. The concentration of lacosamide in each matrix was quantified using a calibration curve prepared from the data of pooled breast milk or plasma. The precision should not be greater than 15%.

To assess dilution integrity, lacosamide in breast milk or plasma at a concentration of 1 μg/mL was diluted 100-fold with blank breast milk or plasma. Furthermore, lacosamide in breast milk or plasma at a concentration of 10 μg/mL was diluted 200-fold with blank breast milk or plasma. Six samples were measured, and the accuracy and precision were determined. The precision and accuracy should not be over 15%.

Lacosamide stability (1.25 and 80 ng/mL) in breast milk and plasma was investigated. The lacosamide remaining 24 h after being stored in breast milk or plasma at 4 °C was quantified. Furthermore, the lacosamide quantity 1 month after being stored in breast milk or plasma at − 30 °C was measured. Freeze–thaw stability was investigated by three freeze–thaw cycles (− 30 °C and room temperature). Six replicates were prepared and analyzed.

### Application of the validated method to clinical samples

This study was approved by the Ethics Committee of the Hokkaido University Hospital (020–0139). The volunteer received a detailed explanation of the study and freely gave her consent to participate. The volunteer was a woman admitted to the Obstetrics Department of Hokkaido University Hospital. She had focal onset epilepsy and had been prescribed levetiracetam and lamotrigine. Because her seizures were could not be controlled by these drugs, lacosamide was introduced several years ago. After the introduction of lacosamide, her seizure frequency was reduced. Because lacosamide could not completely control her seizure, clobazam was additionally prescribed. During the peripartum period, she was administered only lacosamide. The patient delivered at term without complications. This was her second pregnancy. She participated in the study three days after delivery. Although non-compliance is suspected during pregnancy, she certainly took lacosamide at least after delivery. She was orally administrated lacosamide twice a day (100 mg × 2). Before the day she participated in the research, the dosage of lacosamide was increased from 50 mg × 2. Her body weight was 55.72 kg. Plasma samples were obtained 75 min before and 60 min after the oral administration of lacosamide. Breast milk samples were collected 75 min before and after administration (60, 115, 300, and 500 min). The samples were stored at − 30 °C until measurement. The samples were prepared as described in Sample preparation and quantified using UPLC/MS/MS. As the lacosamide concentrations exceeded the calibration range, the samples were diluted 100-fold with drug-free breast milk or plasma.

M/P ratios were expressed as the concentration in breast milk / in plasma. RID was estimated as: RID (%) = (*C*_*milk*_ × *V*_*milk*_ / *D*_*maternal*_) × 100%,

where *C*_*milk*_　is the lacosamide concentration in breast milk; *V*_*milk*_ is the daily volume of milk intake by infants; and *D*_*maternal*_ is the maternal body weight-adjusted lacosamide daily dose in the mother (mg/kg/day).

C_*milk*_ is the average concentration of lacosamide in breast milk (mg/mL) calculated from the area under the curve (AUC_0–12_). AUC_0–12_ was estimated using the linear trapezoidal method. Since we did not obtain the time point 12 h after the last lacosamide administration, the data were compensated by the value at the trough. In the study, *V*_*milk*_ was used average breast milk intake (150 mL/kg/day) for calculation.

In this study, the protein binding of lacosamide in plasma was assessed by quantifying ultrafiltrates using Centrifree® Ultrafiltration Devices (Merck Millipore, Tulagreen, Ireland). The protein binding ratio was expressed as follows: [(total lacosamide concentration – free lacosamide concentration)/total lacosamide concentration] × 100 (%).

## Results and discussion

### Optimization of UPLC/MS/MS condition and sample preparation

Positive ion electrospray tandem mass spectrometry was used to detect lacosamide and its IS (lacosamide-d_3_). Positive ion mass spectra indicated the presence of protonated molecules with *m/z* 251.2 for lacosamide and *m/z* 254.0 for lacosamide-d_3_. The product ions appeared at *m/z* of 108.0, 91.0, 73.9, and 116.0 for lacosamide and *m/z* of 108.0, 91.0, 77.0, and 119.0 for lacosamide-d_3_. The product ion at *m/z* 108.0 had the highest intensity and was used to quantify lacosamide. The transition at *m/z* 254.0 > 108.0 was used to detect lacosamide-d_3_. As a qualifier ion, the transition *m/z* 251.2 > 91.0 was also monitored.

Herein, two types of columns, ACQUITY UPLC BEH C18 column (2.1 × 50 mm, 1.7 µm) and ACQUITY UPLC HSS T3 column (2.1 × 50 mm, 1.8 µm), were preliminarily tested. Since the retention of lacosamide in the HSS T3 column was more than that in the BEH C18 column, we selected the HSS T3 column for lacosamide analysis. As organic solvents, methanol and acetonitrile produced sharp peaks with optimal sensitivity. Herein, methanol was selected as the organic solvent owing to its significant retention in the column. The addition of acid (0.1% acetic acid) did not have significant effects on peak signals or shape. Finally, isocratic elution was performed with 10mM ammonium acetate and methanol (70:30, v/v). The runtime for each sample was 3.5 min.

For sample preparation, simple protein precipitation was selected owing to several advantages, such as simplicity, robustness, and cost efficiency. Finally, 50 µL of breast milk or plasma was required for analysis, and 100 μL of methanol containing the IS was selected as the extraction solvent. Compared with previously described methods for quantifying lacosamide in human plasma [14, 17, 19], the present method has good sensitivity despite the use of a small sample volume. Monfort et al. reported a method for quantifying 19 pharmaceutical drugs, including lacosamide, in breast milk [25]. In their method, 100 μL of breast milk was used for the measurement. A smaller sample volume was required for the present method, and the procedure was simpler compared with their method. Furthermore, the runtime was short for the proposed method.

## Method validation

### Calibration curve

Calibration standard was generated at the concentration range of 0.5–100 ng/mL by spiking eight concentrations of lacosamide to blank breast milk or plasma. In this range, the present method indicated significant linearity for both breast milk (*r*^2^ > 0.997, *n* = 6) and plasma (*r*^2^ > 0.998, *n* = 6). The typical standard curves were y = 0.360759x + 0.0254483 in breast milk and y = 0.350439x + 0.0540461 in breast milk and plasma.

### Accuracy and precision

As indicated in Table 1, the intra- and inter-day precision (R.S.D.) and accuracy (R.E.) were assessed at four concentrations (LLOQ, low, medium, and high). The intra-day R.S.D. ranged from 0.8 to 8.0% and R.E. from − 13.7 to 6.5% for breast milk. The inter-day R.S.D. ranged from 6.5 to 13.4% and R.E. from − 11.7 to 8.4%. The intra-day R.S.D. ranged from 4.2 to 10.6% and R.E. from − 10.2 to 13.1% for plasma. The inter-day R.S.D. ranged from 6.3 to 10.2% and R.E. from − 6.5 to 4.1%. The precision and accuracy met acceptance criteria (< 15%), demonstrating that the method was reproducible.

**Table 1:** Intra- and inter-day reproducibility of lacosamide in human breast milk and plasma

		Intra-day (*n* = 6)			Inter-day (*n* = 6)		
	Spiked (ng/mL)	Found (ng/mL)	R.S.D.^a^ (%)	R.E.^b^ (%)	Found (ng/mL)	R.S.D.^a^ (%)	R.E.^b^ (%)
Breast milk	0.5	0.432	0.8	-13.7	0.442	13.4	-11.7
	1.25	1.32	8.0	5.5	1.30	6.8	4.0
	12.5	13.3	2.7	6.5	13.6	7.6	8.4
	80	78.6	3.7	-1.7	82.4	6.5	2.9
Plasma	0.5	0.449	10.6	-10.2	0.468	10.2	-6.5
	1.25	1.29	4.6	3.3	1.30	6.3	4.1
	12.5	14.1	4.3	13.1	13.0	7.5	4.0
	80	83.9	4.2	4.9	75.9	8.7	-5.1

^a^*R.S.D.* relative standard deviation

^b^*R.E.* relative error

### Matrix effect

To assess the matrix effect, we investigated the precision (R.S.D.) and accuracy (R.E.) at three concentrations (low, medium, and high) in six lots of breast milk and plasma from individual donors. The results are summarized in Table 2. The R.S.D. ranged from 4.1 to 8.0% and R.E. from – 4.2 to 2.9% for breast milk. Further, the R.S.D. of the lots ranged from 1.9 to 4.3% and R.E. from 5.3 to 14.8% for plasma. The data indicated no significant variations among the lots.

**Table 2:** Accuracy and precision in multiple lots

	Spiked (ng/mL)	Found (ng/mL)						Mean (ng/mL)	R.S.D.^a^ (%)	R.E.^b^ (%)
		Lot 1	Lot 2	Lot 3	Lot 4	Lot 5	Lot 6			
Breast milk	1.25	1.38	1.14	1.14	1.31	1.27	1.33	1.26	8.0	0.9
	12.5	12.3	12.9	12.5	13.4	12.5	13.6	12.9	4.1	2.9
	80	69.1	76.5	75.0	77.8	81.0	80.5	76.7	5.7	-4.2
Plasma	1.25	1.55	1.41	1.39	1.44	1.44	1.38	1.44	4.3	14.8
	12.5	13.1	13.4	13.9	14.0	14.4	14.1	13.8	3.5	10.5
	80	82.2	84.4	82.9	83.8	86.0	86.1	84.2	1.9	5.3

^a^*R.S.D.* relative standard deviation

^b^*R.E.* relative error

### Selectivity and carry-over

**Fig. 1:**
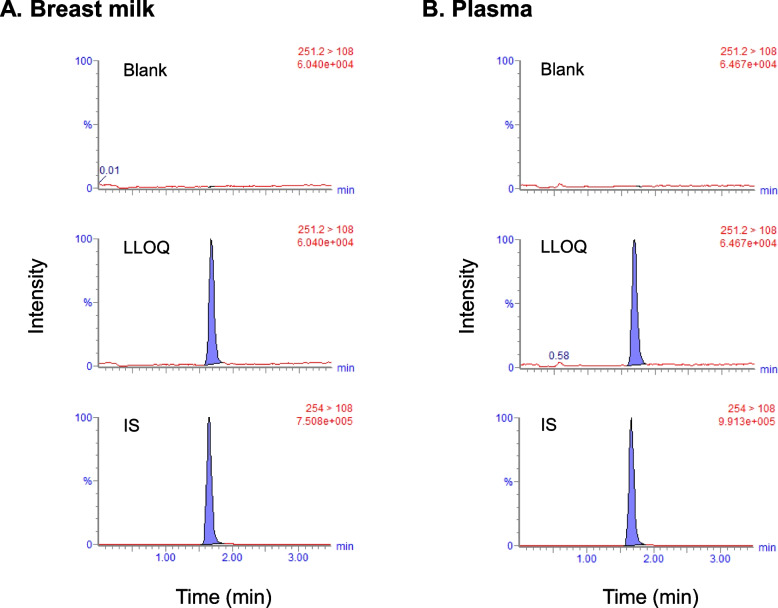
Representative chromatograms of lacosamide in human breast milk (**A**) and plasma (**B**). Chromatograms of blank samples, lower limit of quantitation (LLOQ) of lacosamide (0.5 ng/mL), and internal standard (IS: lacosaide-d_3_) are depicted

To investigate selectivity, six different lots of matrices (breast milk or plasma) with or without the LLOQ level (0.5 ng/mL) of lacosamide and IS were prepared and analyzed. Figure 1 depicts the representative chromatograms of the blank, LLOQ level of lacosamide, and IS in breast milk (A) and plasma (B). No significant interference was detected in the blank breast milk or plasma at the retention time of lacosamide, indicating that the present method has adequate selectivity. Furthermore, carryover was not observed in breast milk or plasma samples.

### Dilution integrity

In the present method, calibration curves ranged from 0.5 to 100 ng/mL for breast milk and plasma. The lacosamide concentration in an authentic clinical sample exceeded the upper limit of quantification in the calibration curve, according to previous reports on plasma (reference range: 5–10 μg/mL) [26]. Therefore, the present method required a dilution process. To assess dilution integrity, lacosmaide in breast milk or plasma at a concentration of 1 μg/mL was diluted 100-fold with drug-free breast milk or plasma (Table 3). Accuracy (R.E.) was found to be 11.3% and precision (R.S.D.) 2.2% for breast milk. The quantification method for plasma indicated an accuracy of 10.5% and precision of 2.7%. We also investigated the dilution integrity to reveal whether the present method can be used for high-concentration samples (Table 3). Lacosamide in breast milk or plasma at a concentration of 10μg/mL was diluted 200-fold with blank breast milk or plasma. Consequently, R.E. was found to be 13.9% and R.S.D. was 4.1% for breast milk. The quantification method for plasma indicated an accuracy of 5.6% and precision of 6.4%. The precision and accuracy met the acceptance criteria (within 15%).

**Table 3:** Dilution integrity

	Sample concentration (μg/mL)	Dilution factor	Nominal concentration (ng/mL)	Found concentration (ng/mL)	R.S.D.^a^ (%)	R.E.^b^ (%)
Breast milk	1	100	10	11.13	2.2	11.3
	10	200	50	57.0	4.1	13.9
Plasma	1	100	10	11.05	2.7	10.5
	10	200	50	52.8	6.4	5.6

^a^*R.S.D.* relative standard deviation

^b^*R.E.* relative error

### Stability

In this study, the short-term (24 h at 4 °C), long-term (1 month at − 30 °C), and freeze–thaw stability in breast milk and plasma at two concentrations (low and high) were investigated. The results are summarized in Table 4. For short-term stability, the deviation from nominal concentration in breast milk ranged from 106.6 to 112.7%, and from 106.0 to 108.1% in plasma. For long-term stability, the mean concentration of lacosamide ranged from 101.9 to 107.9% in breast milk, and from 107.6 to 112.5% in plasma. The remaining lacosamide ranged from 102.2 to 106.5% in breast milk and from 108.2 to 111.3% in plasma after three freeze–thaw cycles. These concentrations were within 15% of nominal concentration, indicating that no significant decomposition occurred.

**Table 4:** Stability of lacosamide in breast milk and plasma

		Stability (% remaining) (Mean ± S.D., *n* = 6)								
	Concentration (ng/mL)	24 h (4 °C)			1 month (− 30 °C)			Freeze–thaw (− 30 °C and room temperature, 3 cycles)		
Breast milk	1.25	112.7	±	9.8	107.9	±	2.4	106.5	±	9.3
	80	106.6	±	6.3	101.9	±	2.9	102.2	±	7.8
Plasma	1.25	108.1	±	7.6	107.6	±	6.9	111.3	±	6.5
	80	106.0	±	6.6	112.5	±	5.9	108.2	±	4.3

### Application of the method to clinical samples

To assess the suitability of the method for clinical samples, we quantified lacosamide in authentic samples obtained from a lactating woman who was prescribed lacosamide for controlling seizures. The patient was orally administered lacosamide twice daily (100 mg × 2). Plasma samples were obtained before (trough) and 60 min after lacosamide administration. Breast milk samples were obtained before and after lacosamide administration. (60, 115, 300, and 500 min). In this study, we collected trough samples 75 min before administration because of the patient’s condition. Generally, trough samples are collected immediately before administration.

**Fig. 2:**
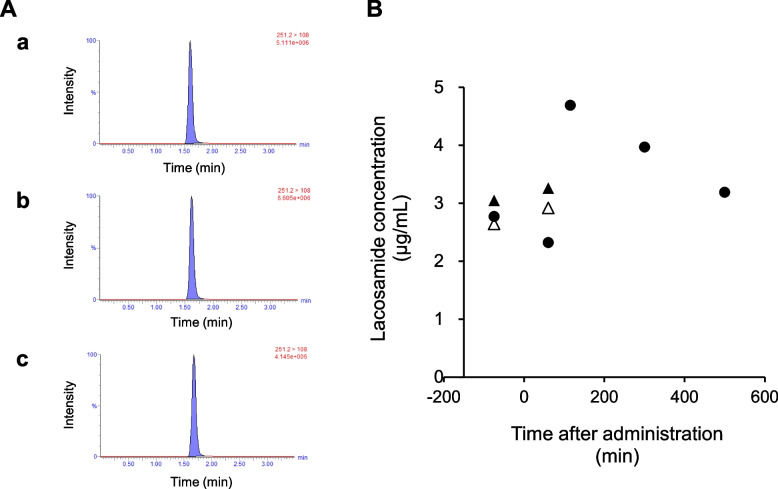
Application to the validated method for quantification of authentic clinical samples. **A** Chromatograms of lacosamide in the patient samples were obtained 60 min after administration: (a) breast milk, (b) plasma, and (c) plasma ultrafiltrates. **B** Graph indicating lacosamide concentration in breast milk (●), plasma (▲), and plasma ultrafiltrate (△; free form of lacosamide). The patient was orally administered lacosamide twice daily (100 mg × 2). Plasma samples were obtained before and 60 min after oral administration of lacosamide. Breast milk samples were collected before and after administration (60, 115, 300, and 500 min).

As depicted in Fig. 2A, lacosamide was detected in both breast milk (a) and plasma (b).

No interfering peaks were detected, and the validated method was suitably applied for detecting lacosamide in the plasma and breast milk of the lactating woman. Plasma concentrations of lacosamide were 3.05 (trough) and 3.26 μg/mL (60min after administration). The breast milk concentrations were 2.77 (trough), 2.32 (60 min after administration), 4.69 (115 min after administration), 3.97 (300 min after administration), and 3.19 μg/mL (500 min after administration). Time profiles are depicted in Fig. 2B. Lacosamide concentrations in breast milk remained high even hours after administration. It has been reported that breast milk levels from a woman took lacosamide (orally 200 mg/day) were 14.27 μM (3.57 μg/mL) at trough, 21.8 μM (5.46 μg/mL) at 2 h after administration, and 16.92 μM (4.24 μg/mL) at 6 h after administration [10]. The milk concentration–time profile was similar to that described in a previous report. Lacosamide has a long half-life (12–14h in young subjects) [27]. In this study, the M/P ratio was calculated to be 0.91 from the trough data. Further, Landmark et al. reported that the mean M/P ratio of lacosamide was 0.83 [13]. Our data were not markedly different from previously reported values, suggesting the suitability of the present method for evaluating the transfer of lacosamide to breast milk. These data suggest that lacosamide is not concentrated in breast milk. Herein, the RID value was found to be 14.6%. As mentioned in Introduction, several case reports are available on RID estimation. Monfort et al. estimated an RID of 29.9% [12]. Kohn et al. reported an RID value of 22% and normal health and development of the infant [11]. However, Ylikiotlia et al. reported a low lacosamide level in milk and RID value of 1.8% [9]. RID greater than 10% of the lowest end of the weight-adjusted maternal or infant dosage may not be safe [28]. Although substantiated cutoff data have not been fully established, a 10% cutoff value has been widely used [28]. The RID value estimated in this study was relatively high (> 10%); therefore, further safety assessments are required. However, breast milk concentrations do not indicate infant pharmacokinetic processes such as absorption, metabolism, and elimination. It has been reported that lacosamide is rapidly absorbed and the absolute bioavailability is 100% [27]. In future studies, directly assessing infant blood concentrations after breastfeeding is warranted. Since the volunteer discontinued breastfeeding 1 month after childbirth at the time of check-up owing to her clinical condition, the effects of breastfeeding on newborns were unclear.

To estimate the plasma protein-binding ratios of lacosamide, ultrafiltrate plasma samples were quantified and analyzed. As depicted in Fig. 2A (c), lacosamide was detected in ultrafiltered plasma samples. The free concentration of lacosamide is plotted in Fig. 2B. The protein binding ratios of lacosamide in plasma were 13.4% (trough) and 10.4% (60 min after administration). The plasma protein binding value was similar to the previously reported value [29]. Generally, the degree of protein binding affects the transfer of drugs to breast milk. Generally, the concentrations of plasma albumin and protein-binding drugs vary during pregnancy and after delivery [30]. Because this study evaluated only 1 analyte and 1 case, a detailed discussion is not currently possible. The present method can be used to estimate the protein binding of lacosamide in lactating women. Although protein binding of lacosamide was relatively low, future studies with larger volunteers can be performed to investigate free concentrations and protein binding of lacosamide and the effects of the degree of passage into breast milk.

This clinical study had some limitations. First, it is a case report. Since the study aimed to develop a method for quantifying lacosamide in breast milk and plasma, we obtained only a single sample. Second, the M/P ratios were calculated at a single time point because collection of blood samples multiple times was a burden on the patient. Notably, M/P ratio should be expressed as AUC ratio [28]. Further studies with a large sample size are needed to clarify the exposure level of lacosamide via breast milk and the occurrence of adverse effects.

## Conclusions

Herein, we developed a simple and robust UPLC-MS/MS method for quantifying lacosamide in human breast milk and plasma. Only 50 µL of breast milk and plasma were used, and the sample preparation involved simple protein precipitation. Chromatography was performed under simple isocratic conditions, and the run time was short (3.5 min). This method was suitably applied for lacosamide quantification in clinical samples. We determined the concentrations of lacosamide in breast milk, plasma, and ultrafiltrate plasma, which were donated by a volunteer who was regularly administered lacosamide. The present method can be useful for facilitating future studies investigating the safety of lacosamide administration during breastfeeding and its transfer to breast milk.

## Data Availability

All data generated or analyzed during this study are included in this published article.

## Abbreviations

AUC: Area under the curve
IS: Internal standard
LLOQ: Lower limit of quantification
M/P: Milk/plasma
MRM: Multiple reaction monitoring
R.E.: Relative error
RID: Relative infant dose
R.S.D.: Relative standard deviation
UPLC/MS/MS: Ultra-performance liquid chromatography tandem mass spectrometry
